# Large-Scale Brain Functional Network Integration for Discrimination of Autism Using a 3-D Deep Learning Model

**DOI:** 10.3389/fnhum.2021.687288

**Published:** 2021-06-02

**Authors:** Ming Yang, Menglin Cao, Yuhao Chen, Yanni Chen, Geng Fan, Chenxi Li, Jue Wang, Tian Liu

**Affiliations:** ^1^The Key Laboratory of Biomedical Information Engineering of Ministry of Education, Institute of Health and Rehabilitation Science, School of Life Sciences and Technology, Xi’an Jiaotong University, Xi’an, China; ^2^National Engineering Research Center for Healthcare Devices, Guangzhou, China; ^3^The Key Laboratory of Neuro-informatics and Rehabilitation Engineering of Ministry of Civil Affairs, Xi’an, China; ^4^Xi’an Children’s Hospital, Xi’an, China

**Keywords:** autism spectrum disorder, functional MRI, convolutional neural network, brain functional network, classification

## Abstract

**Goal:**

Brain functional networks (BFNs) constructed using resting-state functional magnetic resonance imaging (fMRI) have proven to be an effective way to understand aberrant functional connectivity in autism spectrum disorder (ASD) patients. It is still challenging to utilize these features as potential biomarkers for discrimination of ASD. The purpose of this work is to classify ASD and normal controls (NCs) using BFNs derived from rs-fMRI.

**Methods:**

A deep learning framework was proposed that integrated convolutional neural network (CNN) and channel-wise attention mechanism to model both intra- and inter-BFN associations simultaneously for ASD diagnosis. We investigate the effects of each BFN on performance and performed inter-network connectivity analysis between each pair of BFNs. We compared the performance of our CNN model with some state-of-the-art algorithms using functional connectivity features.

**Results:**

We collected 79 ASD patients and 105 NCs from the ABIDE-I dataset. The mean accuracy of our classification algorithm was 77.74% for classification of ASD versus NCs.

**Conclusion:**

The proposed model is able to integrate information from multiple BFNs to improve detection accuracy of ASD.

**Significance:**

These findings suggest that large-scale BFNs is promising to serve as reliable biomarkers for diagnosis of ASD.

## Introduction

Autism spectrum disorder (ASD) represents a complex developmental disorder characterized by social deficits and restrictive or repetitive behaviors. Unlike other fields of medicine, psychiatry disorders lack valid physiological diagnostic criteria based on validated biomarkers (American Psychiatric Association, 2013). Mostly, ASD is diagnosed by subjective judgment of clinical symptoms and behaviors by clinicians. However, these methods require doctors to have high level of professional knowledge, and the diagnosis results are susceptible to doctors’ subjectivity. To find a more objective biomarker for identification of ASD, many researchers focus on deriving effective biomarkers such as genetics, epigenetics, body metabolism, and neuroimaging (Goldani et al., 2014). Neuroimaging is regarded as a promising non-invasive technique to uncover latent patterns of human brains. A human brain can be modeled as a complex system with various regions performing different structures and functions by using structural magnetic resonance imaging (sMRI), functional magnetic resonance imaging (fMRI), and positron emission tomography (PET) et.al. Previous neuroimaging studies have revealed alternation in both structural and functional connectivity of the brain among neurological or psychiatric disease populations (Mueller et al., 2013). Among all kinds of examination approaches, fMRI, especially resting state fMRI (rs-fMRI) recoding the changes of blood oxygen level-dependent (BOLD) signals, has been widely used for investigating mental disorders such as Alzheimer’s disease (Qureshi et al., 2019b), schizophrenia (Yan et al., 2019), and ASD (Abraham et al., 2017).

Functional magnetic resonance imaging data is organized in a 4-D matrix format with high dimensionality (∼1 million) containing both spatial and temporal information. This makes it a difficult task to directly utilize the original data as input for classification algorithms. To address the high dimensionality of the data, many dimensionality reduction technologies have been proposed (Abdi and Williams, 2010; Suk et al., 2015; Soussia and Rekik, 2018). Instead of using original fMRI data, some have proposed brain function network analysis characterizing the “relationship” between regions of interest (ROIs). Based on the fact that the cerebral blood flow refreshes the neural activity across regions of the brain, modeling functional connectivity (FC) is helpful for understanding the neural basis of mental disorder (Lindquist, 2008). The most commonly used FC model is Pearson’s correlation, which can be calculated using BOLD signals between two brain regions. A brain functional network (BFN) is constructed based on the strength of the FC of all locations predefined by an atlas. BFN construction approaches explicitly reduced the dimensionality from 4-D into a 1-D vector. Many machine learning (ML) methods have been successfully employed in the automated classification of altered BFNs related to ASD (Uddin et al., 2013; Abraham et al., 2017). Some methods adopted sparse methods to implement implicit dimension reduction by adding an extra sparse regularization term [e.g., Lasso (Tibshirani, 1996) or the Elastic Net (Zou and Hastie, 2005)] to the loss function.

However, the commonly used correlation for describing FC between ROIs just captures a linear relationship and is not suitable for characterizing high-order or non-linear features (Shojaee et al., 2019). Furthermore, collapsing the data into a feature vector (Vectorization) discards the spatial information of the brain regions (Kong et al., 2019). In addition, traditional classification algorithms such as support vector machine (SVM) (Cortes and Vapnik, 1995), Random Forest (Liaw and Wiener, 2002), and Naive Bayes (Rish, 2001) belong to a shallow model, which limits their capacity to extract the structural information hidden in BFNs.

To derive BFNs with both functional connectivity and spatial information, independent component analysis (ICA) has been widely used (Rajapakse et al., 2006; Nickerson et al., 2017). ICA is a pure data-driven method, which can generate highly reproducible large-scale brain networks (Damoiseaux et al., 2006). However, the ICA components do not correspond exactly to meaningful brain networks. Usually, these components will be inspected by experienced clinicians or researchers to remove artifact components. Here, we use an automatic clustering algorithm (Beckmann et al., 2009; Filippini et al., 2009) to perform the selection of ICA components. The selected meaningful components can be used for deriving each subject’s subject-specific spatial maps by dual regression. Each individual spatial map captures variabilities in both the shape and amplitude of the corresponding resting state network between groups (Nickerson et al., 2017). This kind of feature representation in BFNs employs a 3-D functional rs-fMRI modality, which contains more information than FC coefficients.

Recently, deep learning has gained much attention in various computer vision tasks (Redmon et al., 2016; Krizhevsky et al., 2017; Ledig et al., 2017). Deep learning has also been applied in medical image analysis, such as lesion segmentation (Isensee et al., 2018), MRI reconstruction (Schlemper et al., 2018), and registration (Yang et al., 2017). The deep neural network (DNN) is powerful for its capability to directly learn useful features from raw data and eliminate the need to manually design features in many other machine learning algorithms. Several studies have used stacking auto-encoders to build a DNN for classification of brain disorders such as Alzheimer’s disease (Suk et al., 2015), autism (Heinsfeld et al., 2018) and schizophrenia (Kim et al., 2016). In addition, recurrent neural network (RNN) and graph convolutional neural network (GCN) are also used in the early diagnosis of mental diseases. Dvornek et al. (2017) used long short-term memory (LSTM) to classify the time series data of resting-state fMRI, and the accuracy of cross-validation reached 68.5%. Yan et al. (2019) designed a multi-scale convolutional neural network-gated recurrent unit (CNN-GRU) model to learn the time series obtained from ICA and realized the classification of schizophrenia based on multi-site data. Ktena et al. (2017) took advantage of the spectral method of GCN to learn the similarity of brain functional connectivity networks and applied it to the early diagnosis of ASD. Unlike the methods mentioned above, 3-D convolutional neural network (3-D CNN) takes 3-D images as input rather than FC vector or time series data, capturing hierarchical features by integrating multi scales of features with different layers for spatial pattern representation and recognition (LeCun et al., 2015). In recent years, 3-D CNN has been successfully used in the classification of Alzheimer’s disease (Qureshi et al., 2019b), schizophrenia (Qureshi et al., 2019a), and early MCI (Kam et al., 2018), which achieved competitive performance of approximately 74–98% accuracy compared to traditional machine learning algorithms. Nevertheless, to date, 3-D CNN has not been used to classify large-scale BFNs in ASD, and the abnormal organization of the large-scale BFN in ASD subjects is not well understood. For these reasons, in the present study, we take the group ICA features as input and build a 3-D CNN architecture to model the differences in both “shape” (e.g., spatial activation patterns) and amplitude (e.g., the magnitude of the BOLD activity) of one or more BFNs, and we investigate both intra- and inter-BFN association changes to find a reliable and objective biomarker for diagnosis.

The contributions of our work can be summarized in three aspects. (1) We used group ICA and dual regression to derive 3-D functional network spatial maps as potential biomarkers for identification of ASD. (2) We developed a variant of the VGG network, which involves channel attention mechanism, to integrate both intra- and inter-BFN association changes to find a reliable and objective biomarker for diagnosis. (3) A systematic comparison of our method with traditional machine learning framework has also been implemented. Our proposed model can improve detection accuracy of ASD.

## Materials and Methods

**FIGURE 1:**
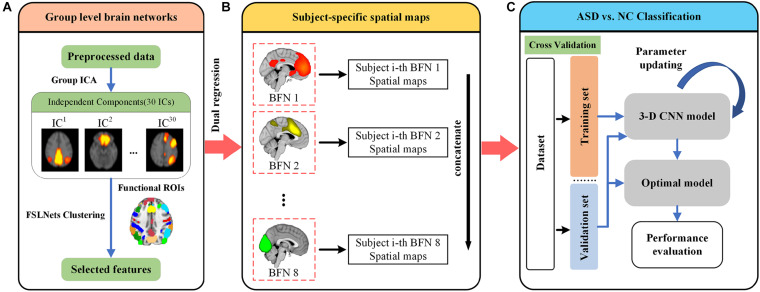
Illustration of the proposed framework for ASD diagnosis. **(A)** All rs-fMRI data were preprocessed using the C-PAC pipeline. The independent components were derived using Group-ICA, and further inspected to identify eight well defined brain function networks (BFNs). **(B)** The selected BFNs were used to extract subject-specific spatial maps using dual regression. Eight subject-specific spatial maps were then concatenated together as input to the classifier. **(C)** The data were randomly split into training and validation sets. A total of 10-fold cross validation strategy was used for evaluating classification performance. The details of 3-D CNN model will be introduced later.

Figure 1 illustrates the overall framework for discrimination of ASD. We first preprocessed all rs-fMRI data from the NYU site using the C-PAC pipeline. The preprocessed data were used to perform group ICA to generate 30 independent components. The good components were filtered by clustering and template matching, which would be used for further analysis (Figure 1A). Second, we took the selected components to perform dual regression for each subject. Eight subject-specific spatial maps were generated and concatenated for each subject, which would be used as input for classification (Figure 1B). We then randomly split all subjects into training and validation sets. The subject-specific spatial maps were fed into a 3-D CNN model. Finally, a 10-fold cross validation strategy was adopted for the classification performance evaluation (Figure 1C).

### Dataset

We ran our study on the Autism Brain Imaging Data Exchange (ABIDE) (Di Martino et al., 2014), which is a publicly available multi-site data repository. The first phase of ABIDE (ABIDE-I) compiles a dataset of 1112 individuals from 17 sites and consists of 539 individuals with ASD and 573 typical controls. Since the scan parameters vary across sites, ABIDE is a highly heterogeneous dataset. To avoid the impact of data heterogeneity, we collected raw rs-fMRI data from the whole NYU site, consisting of 79 ASD subjects and 105 normal controls (NCs). Table 1 presents the complete demographic of the selected data.

**TABLE 1:** Demographics characteristics of the selected subjects.

	Autism spectrum disorder	Normal control	*p*-Value
	*N* = 79; 11 F/68 M	*N* = 105; 26 F/79 M	
Age	14.52 ± 6.97	15.81 ± 6.25	0.039
Full IQ	107.91 ± 16.62	113.15 ± 13.12	0.022
Verbal IQ	105.81 ± 16.13	113.13 ± 12.60	0.001
Performance IQ	108.81 ± 17.42	110.06 ± 13.67	0.600
ADOS total	11.30 ± 4.08		
ADOS communication score	3.54 ± 1.55		
ADOS social score	7.76 ± 2.97		

*ADOS, autism diagnostic observation schedule.*

#### Structural Data Acquisition

All participants were scanned with a Siemens 3 Tesla Allegra scanner. An MPRAGE sequence was used with the following parameters: TR/TE/TI = 2530/3.25/1100 ms, flip angle = 7°, FoV read = 256 mm, slice thickness = 1.33 mm, voxel size = 1.3 × 1.0 × 1.3 mm, matrix = 256 × 256 × 171, bandwidth = 200 Hz/Px, and total scan time = 8:07 min.

#### Functional Data Acquisition

Resting state functional MRI data were acquired using a 2-D echo-planar imaging (EPI) acquisition type with the following parameters: TR/TE = 2000/15 ms, flip angle = 90°, FoV read = 240 mm, slice thickness = 4 mm, voxel size = 3.0 × 3.0 × 4.0 mm, bandwidth = 3906 Hz/Px, and total scan time = 6.00 min. During the rs-fMRI scan, most participants were asked to relax with their eyes open and look at a cross-hair on a black background screen. Data were also included for some participants who were asked to keep their eyes closed.

### Pre-processing of Functional MRI Data

We preprocessed the data using a widely adopted pipeline called Configurable Pipeline for the Analysis of Connectomes (C-PAC) (Craddock et al., 2013), which involves skull striping, slice timing correction, realignment to correct for motion, and bandpass filtering (0.01–0.1 Hz). The functional images were smoothed with a 5-mm full width half maximum (FWHM) Gaussian kernel, registered to a standard anatomical space (MNI152) and resampled to 4 mm.

#### Independent Component Analysis

We used FSL Multivariate Exploratory Linear Optimized Decomposition into Independent Components (MELODIC^1^) version 3.15 to perform group ICA. Preprocessed data were concatenated and entered into a group ICA to identify large-scale functional networks across the population. Usually, more than 20 components are necessary for identification of meaningful components, whereas model orders >100 showed a decrease in ICA repeatability (Abou-Elseoud et al., 2010). The number of independent components was set to 30. Variance normalization and thresholding were further used to fit a Gaussian and 2 Gamma mixture model to the intensity histogram of the Z-transformed IC spatial maps.

**FIGURE 2:**
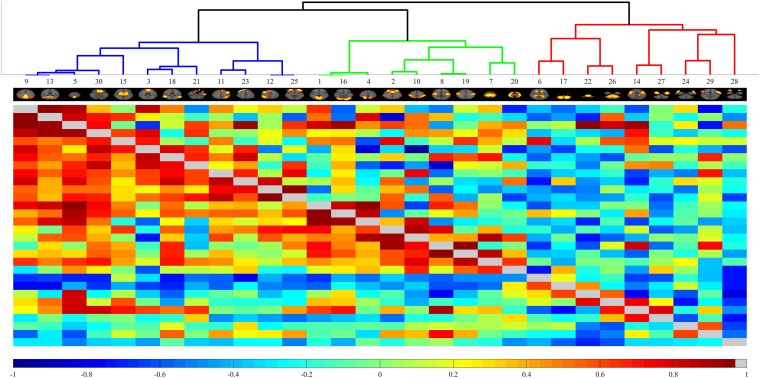
Automated clustering dendrograms of the independent component-based brain functional networks acquired through Melodic ICA. The right branch depicted in red line represents noise or artifacts. The left branch contains good components that will be further inspected *via* power spectrum.

Finally, MELODIC yielded 30 independent component maps with the local false-discovery rate *p* < 0.5. Among the 30 independent components, we used an automatic clustering tool, FSLNets^2^, to divide these independent components into good networks and noise and/or artifacts. Second, we inspected the power spectra graph for each good independent component. The power spectrum of a typical network is in the low-frequency range, whereas an artifact power spectrum shows a multipeak pattern in the 0–0.1 Hz range. Furthermore, we used an FSL utility, *fslcc*, to spatially correlate all 15 good components derived by FSLNets to some set of reference networks. The reference networks we used are from the Stanford Functional Imaging in Neuropsychiatric Disorders Lab^3^ (Shirer et al., 2012). The names of reference networks are shown in Table 2. Finally, eight functional networks were filtered as the output of group ICA. Figure 2 shows all 30 components. The dendrogram contains two major branches representing good functional networks (in blue and green color) and noise and/or artifacts (in red color). All these functional networks served as templates to generate subject-specific brain function networks in the next step.

**TABLE 2:** The names of reference networks.

Number	Reference network
1	Anterior insula/dorsal ACC (anterior salience network)
2	Auditory network
3	Basal ganglia network
4	PCC/MPFC (dorsal default mode network)
5	Higher visual network
6	Language network
7	Left DLPFC/parietal (left executive control network)
8	Sensorimotor network
9	Posterior insula (posterior salience network)
10	Precuneus network
11	Primary visual network
12	Right DLPFC/parietal (right executive control network)
13	Retrosplenial cortex/medial temporal lobe (ventral default mode network)
14	Intraparietal sulcus/frontal eye fields (visuospatial network)

#### Dual Regression

To evaluate individual differences in BFNs, we applied the dual regression (Beckmann et al., 2009; Nickerson et al., 2017) approach in FSL v6.0.1^4^. This method has been widely used in comparing large-scale BFNs between groups (Filippini et al., 2009; Nickerson et al., 2017). Dual regression decomposes each subject’s 4-D fMRI data into a set of spatial maps and corresponding time courses. Dual regression is an effective approach to investigate network shape and amplitude in functional connectivity analyses (Nickerson et al., 2017). Dual regression analysis proceeds in two steps. First, for each subject, a set of template networks is regressed into the subject’s 4-D spatial-temporal dataset. This results in a set of subject-specific time courses, corresponding to each IC template. Second, the network-specific time courses are used as predictors in a multivariable multiple linear regression into the same 4-D dataset, resulting in a set of subject-specific 3-D spatial maps. The template network can be derived using FSL’s group ICA, from an atlas, or using functional localizers. Based on these 3-D spatial maps of BFNs for each subject, we build a 3-D CNN model to learn sophisticated feature representation for classification.

### 3-D CNN Architecture for Classification

In the present study, we implemented stratified 10-fold cross validation to evaluate the effectiveness of our algorithm. This method of splitting data ensures that the proportion of ASDs and NCs was the same across all folds.

#### Backbone Structure

**FIGURE 3:**
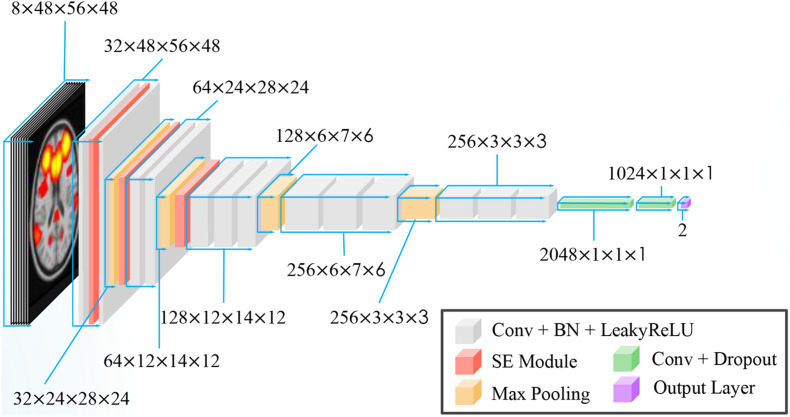
Modified VGG-Net 3-D CNN architecture with SE module integrated.

A variant of VGG-net (Simonyan and Zisserman, 2014) was used for our task. VGG-net is a popular convolution network model that has been used in many studies (Qureshi et al., 2019a,b; Duc et al., 2020). Instead of using 2-D convolution in vanilla VGG, 3-D convolution was adopted in our network. Batch Normalization (Ioffe and Szegedy, 2015) and Leaky Rectified Linear Unit (LeakyReLU) (Maas et al., 2013) were used as activation functions. Because CNN is a highly non-convex function, poor initialization strategy may induce the CNN into a local sub-optimal solution, resulting in bad generalization performance. To alleviate this problem, we initiated our 3-D CNN with the initialization strategy proposed by He et al. (2015). The overview of our proposed CNN is shown in Figure 3.

#### Channel Attention Module

Vanilla VGG achieved good performance in image classification by stacking a series of convolution layers with the same kernel size of 3 × 3 × 3 and interleaved with non-linear activation and max-pooling layers. This kind of hierarchical architecture can capture local spatial patterns along all input channels. To model the independencies between channels, channel switching, combination (Zhang et al., 2017, 2018) or using reinforcement learning to reorganize the network paths (Ahmed and Torresani, 2017), channel-wise attention was proposed to bias the allocation of resources to the most informative channels (Hu et al., 2018). The squeeze and excitation (SE) module is an efficient component that can be added to any CNN model easily. The key point of SE module is that it can calibrate the feature map and emphasize important channels by learning a channel-specific descriptor. We integrated several SE blocks into the variant VGG structure to boost classification performance.

**FIGURE 4:**
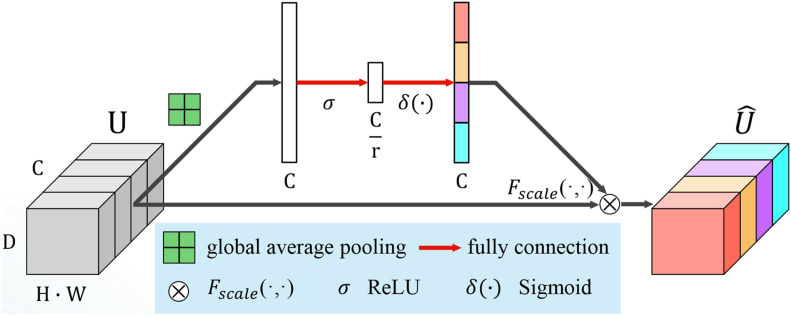
Architecture of squeeze and excitation (SE) module.

The SE module consists of two parts, *squeeze* and *excitation.* A diagram of an SE building block is depicted in Figure 4. Let us assume that U = [u_1_, u_2_,…,u_F_] is an input feature map, where u_*f*_ϵ*ℝ*^H×W×D^ is a single channel with size *H* × *W* × *D*. *H*,*W* and *D* are the spatial height, width, and depth, respectively. Channel squeeze is performed *via* a global average pooling layer, productor vector **z**ϵ*ℝ*^1×1×1×*F*^, with the *f-*th element given by:

(1) $${\text{z}}_{\text{f}}=\frac{1}{\text{H}\times \text{W}\times \text{D}}\,\sum_{\text{h}=1}^{\text{H}}\sum_{\text{w}=1}^{\text{W}}\sum_{\text{d}=1}^{\text{D}}{\left[{\text{u}}_{f}\right]}_{i,j,k}$$

This operation embeds the global spatial information into vector **z**. To make full use of the information hidden in **z**, an excitation operation is designed. Two fully connected layers, the ReLU (Nair and Hinton, 2010) function δ and a sigmoid activation function σ(⋅), are used to transform **z**:

(2) $$\text{s}=\sigma \,\left({\text{W}}_{1}\,\delta \,\left({\text{W}}_{2}\,z\right)\right)$$

where $W_{1}\,\epsilon \,{\mathbb{R}}^{\frac{F}{r}\times F}$, $W_{2}\,\epsilon \,{\mathbb{R}}^{F\times \frac{F}{r}}$ and r is the reduction ratio to limit the model complexity and aid generalization. **s** encodes the channel-wise dependencies and is used to excite or recalibrate U to:

(3) $$\hat{U}=F_{s\,c\,a\,l\,e}\,\left(u_{f},s_{f}\right)=s_{f}\cdot u_{f},\text{for}\,\text{f}=1,2,\cdots ,\text{F}$$

where $F_{s\,c\,a\,l\,e}\,\left(u_{f},{\hat{z}}_{f}\right)$ for the channel-wise multiplication between the feature map u_*f*_ϵ*ℝ*^H×W×D^ and the scalar *s_f_*. The importance of the *i*-th channel is indicated by *s*_*f*_ ∈ [0,1].

Here, we inserted three SE blocks into the 3-D VGG net. The reduction ratio was set to 16. Details of our model are presented in Table 3.

**TABLE 3:** Details of the proposed MCSE-VGG architecture.

Layer	Feature map	Stride	Kernel	Activation structure
Convolution	32	1 ×1 ×1	3 ×3 ×3	Conv
**SE block**				
Convolution	32	1 ×1 ×1	3 ×3 ×3	BN + LReLU + Conv
Max-pooling		2 ×2 ×2	2 ×2 ×2	
**SE block**	32			
Convolution	64	1 ×1 ×1	3 ×3 ×3	BN + LReLU + Conv
Convolution	64	1 ×1 ×1	3 ×3 ×3	BN + LReLU + Conv
Max-pooling	64	2 ×2 ×2	2 ×2 ×2	
**SE block**				
Convolution	128	1 ×1 ×1	3 ×3 ×3	BN + LReLU + Conv
Convolution	128	1 ×1 ×1	3 ×3 ×3	BN + LReLU + Conv
Convolution	128	1 ×1 ×1	3 ×3 ×3	BN + LReLU + Conv
Max-pooling		2 ×2 ×2	2 ×2 ×2	
Convolution	256	1 ×1 ×1	3 ×3 ×3	BN + LReLU + Conv
Convolution	256	1 ×1 ×1	3 ×3 ×3	BN + LReLU + Conv
Convolution	156	1 ×1 ×1	3 ×3 ×3	BN + LReLU + Conv
Max-pooling		2 ×2 ×2	2 ×2 ×2	
Convolution	256	1 ×1 ×1	3 ×3 ×3	BN + LReLU + Conv
Convolution	256	1 ×1 ×1	3 ×3 ×3	BN + LReLU + Conv
Convolution	256	1 ×1 ×1	3 ×3 ×3	BN + LReLU + Conv
Convolution	2048	1 ×1 ×1	3 ×3 ×3	Dropout + Conv + LReLU
Convolution	1024	1 ×1 ×1	1 ×1 ×1	Dropout + Conv + LReLU
Dense	2			

*MCSE-VGG, multi channel squeeze and excitation VGG-net; LReLU, Leaky Rectified Linear Unit.*

### Performance Evaluation

For performance evaluation, we adopted accuracy (ACC), precision, recall, and F1 score as quantitative metrics. TP, TN, FP, FN, PPV, and NPV denote true positive, true negative, false positive, false negative, positive predictive value, and negative predictive value, respectively. These metrics are defined as below:

$$A\,C\,C=\frac{T\,P+T\,N}{T\,P+T\,N+F\,P+F\,N}$$

$$P\,r\,e\,c\,i\,s\,i\,o\,n=\frac{T\,P}{T\,P+F\,N}$$

$$R\,e\,c\,a\,l\,l=\frac{T\,N}{T\,N+F\,P}$$

$$F\,1=\frac{2\times \text{Precision}\times \text{Recall}}{P\,r\,e\,c\,i\,s\,i\,o\,n+R\,e\,c\,a\,l\,l}$$

## Results

### Large-Scale Brain Functional Networks

**FIGURE 5:**
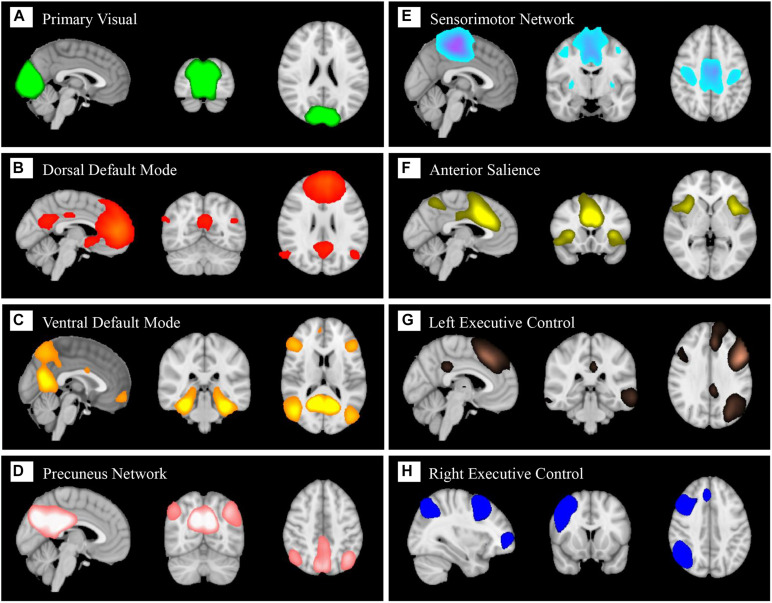
Eight large-scale resting state brain functional networks derived using group independent component analysis (gICA): primary visual network **(A)**, dorsal default mode **(B)**, ventral default mode **(C)**, precuneus network **(D)**, sensorimotor network **(E)**, anterior salience **(F)**, left central executive **(G)**, right central executive **(H)**.

In total, eight components (Figure 5) were identified as BFNs from group ICA. The eight selected resting state functional networks included the primary visual network (PVN), dorsal default mode network (dDMN), ventral default mode network (vDMN), precuneus network (PCUN), sensorimotor network (SMN), anterior salience network (SN), left central executive network (LCEN), and right central executive network (RCEN). They were used to perform dual regression to generate subject-specific time courses for connectivity analysis and spatial maps for classification.

### Network Configuration

We applied PyTorch (Paszke et al., 2019) 1.0 framework to implement our model. Training and testing of this model used one NVIDIA RTX 2080Ti graphical processing unit (GPU). During the training phase, an ADAM (Kingma and Ba, 2014) optimizer was used with an initial learning rate of 0.0005. The initialization strategy proposed by He et al. (2015) was adopted to initiate the 3-D CNN model. The learning rate was decayed by 0.1 if the validation loss did not decrease after 10 epochs. The batch size was set to 12, and the negative slope LeakyReLU was set as 0.01. We used L2 norm regularization on the convolution kernel parameters with a weight of 1e-5. Dropout was set as 0.7. The cross-entropy loss function is applied to the output units to predict the probability of a subject belonging to the NC or ASD group. The results of the validation set of each fold will be shown in the next section.

### Performance Evaluation

#### Baseline

First, we padded subject-specific spatial maps of each BFN into 48 × 56 × 48 and then concatenated them into a 4-D tensor of 8 × 48 × 56 × 48 for each subject. The first dimension represents the number of selected BFNs. All eight selected components are associated with high-level recognition functions, which include the primal visual network, PCUN, default mode network (DMN), SN, executive control networks and SMN. We took this 4-D tensor as input to train a vanilla VGG model as baseline.

#### Channel Attention Based 3-D CNN

**FIGURE 6:**
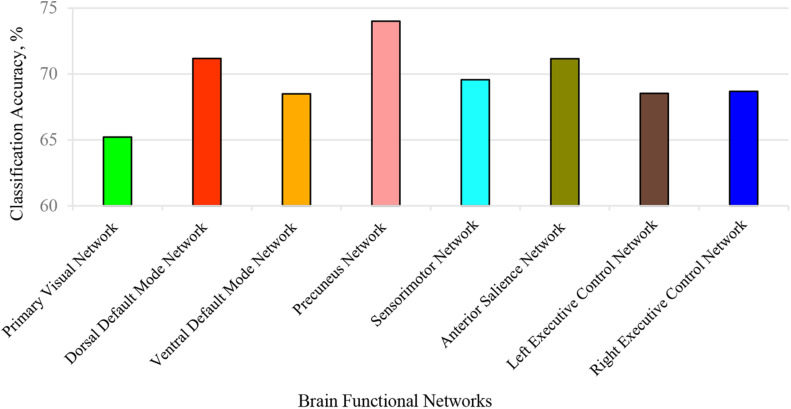
Classification accuracy for each identified brain function network (BFN). Each BFN spatial map was used as input features and fed into SCSE-VGG model for classification. The precuneus network achieved the highest accuracy at 74%.

Here, we conducted two kinds of 3-D CNN integrated with the SE module. One consists of taking each BFN separately into SE-VGG to construct a single channel SE-VGG (SCSE-VGG) diagnosis model. Figure 6 shows the ability to discriminate ASD from NC based on spatial maps corresponding to each of the 8 functional networks for each subject using SE-VGG. Another option is to concatenate 8 BFNs as multi-channel input to SE-VGG (MCSE-VGG). In Table 4, we summarize the performance of our proposed method. Our experiments showed that the MCSE-VGG model outperformed other models by achieving a mean accuracy of 77.74%, which significantly boosted the performance of the baseline model by ∼8%. Compared to various SCSE-VGG models using only one BFN, the proposed MCSE-VGG improved the accuracy by ∼4–12%. The results showed that our proposed method could effectively capture the relationship within multiple BFNs. In addition, the SE block could assist the 3-D CNN learning from multiple BFNs and effectively model the channel-interdependencies information. Among all 8 SESE-VGG models, SESE-VGG with the PCUN, dDMN, and SN showed higher accuracy than those with other BFNs. This indicated that these three networks might play an important role in the development of ASD.

**TABLE 4:** Classification results comparison of different network architectures using 10-fold cross-validation.

Method	Functional network	ACC (%)	Precision (%)	Recall (%)	F1 score
Baseline	All	69.58	69.43	65.54	67.43
SCSE-VGG	PVN	65.21	68.10	50.54	50.02
	PCUN	74.01	71.76	77.32	74.44
	dDMN	71.18	73.38	68.57	70.89
	vDMN	68.49	64.22	65.54	64.87
	LCEN	68.53	70.67	56.96	63.08
	RCEN	68.68	66.62	89.82	76.50
	SN	71.16	77.06	61.96	68.69
	SMN	69.57	71.19	64.82	67.86
MCSE-VGG	All	77.74	76.74	78.57	75.33

*The baseline and MCSE-VGG models use all the eight brain functional networks. MCSE-VGG and SCSE-VGG models are inserted with SE module. SCSE-VGG models are evaluated for each brain functional network.*

#### Compared Methods

We compared the proposed SCSE-VGG with several state-of-the-art methods including both traditional machine learning algorithms and deep learning methods. We extracted the mean time series for ROI defined by the CC200 functional parcellation atlas of the brain. The functional connectivity was calculated by Pearson correlation between each pair of brain regions and generated a correlation matrix. The upper triangle values of the correlation matrix were vectorized as features and fed into an Autoencoder and Multilayer Perceptron (AE-MLP) model. We modified the code from Heinsfeld et al. (2018) and implemented three algorithms, e.g., deep neural network (Heinsfeld et al., 2018), SVM (Cortes and Vapnik, 1995) and random forest (RF) (Liaw and Wiener, 2002), on the NYU dataset. Bengs et al. (2020) used the 4-D fMRI image data and modeled the spatial-temporal information by 3-D convolutional GRU and 3-D CNN. We reported their experimental results on the NYU dataset. We show the results from these models in Table 5.

**TABLE 5:** Performance comparison of the proposed and previous methods.

Method	ACC (%)	F1 score
AE-MLP	68.56	73.87
SVM	62.97	74.24
Random forest	60.62	71.37
convGRU-CNN3D (Bengs et al., 2020)	67.00	71.00
VGG (ours)	69.58	67.43
MCSE-VGG (ours)	77.74	75.33

*The results of convGRU-CNN3D were reported on the paper (Bengs et al., 2020).*

Deep learning-based classification frameworks (including AE-MLP, convGRU-CNN3D, and our proposed model) showed better performance than traditional machine learning-based frameworks (including SVM and RF). The results showed that our proposed model (MCSE-VGG) outperformed other models by achieving a mean accuracy and F1 score of 77.74 and 75.33%, respectively, which surpassed previous studies by ∼9–17 and ∼1–8%. This improvement may be due to the ability of 3-D CNN to make use of the features hidden in both inter- and intra-BFN. 3-D convolution kernel is powerful for extracting spatial information within each BFN, and the channel attention mechanism can further recalibrate the importance of spatial features extracted by convolution layers.

### Inter-Network Connectivity Analysis

Although our experiments have shown good performances compared to existing methods, the deep learning framework lacks interpretability. To further validate the effectiveness of our selected BFNs to serve as reliable imaging biomarkers for ASD diagnosis, we performed an inter-network analysis on the basis of the dual regression results using the FSLNets package^5^. The time courses derived by stage one of the dual regression were used as input for the modeling network. Artifactual components were regressed out over the corresponding time courses, and only the selected eight components were preserved and considered for subsequent statistical analysis. We took ridge regression partial correlation (PC) as a measure of direct connections (Smith et al., 2011) between each pair of components. The correlation coefficients were then transformed into *Z*-values *via* the standard Fisher’s transform. The PC matrices were used as input to the general linear model (GLM) analysis, and an unpaired nonparametric test with 5000 permutations was run to test differences in connection strength between ASD and NC.

**FIGURE 7:**
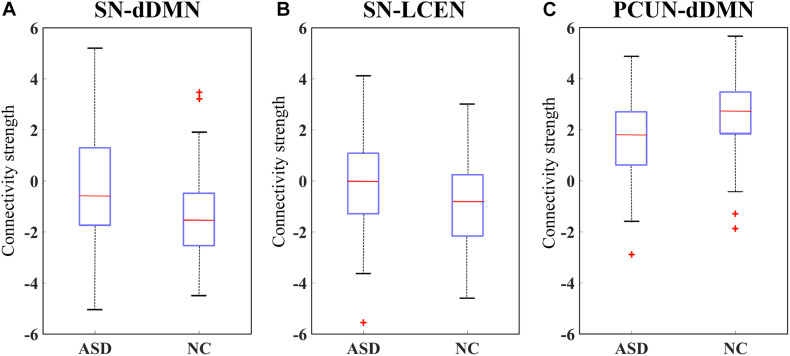
Box plot of the connectivity differences in ASD versus NC. Connectivity strength is shown for the brain functional networks (BFN) between **(A)** anterior salience network (SN) and dorsal default mode network (dDMN) and **(B)** SN and left central executive network (LCEN), and **(C)** precuneus network (PCUN) and dDMN.

We selected the most significant network edges (*p* = 0.05) from the GLM output and created boxplots for the two groups as illustrated in Figure 7. As a result, the strengths of connectivity were significantly increased between the SN and dDMN in ASD patients compared to controls. A similar result was also found between the SN and LCEN. Furthermore, a reduced connective strength was obtained between the PCUN and dDMN.

## Discussion

In this study, we proposed a novel framework to model inter- and intra-associations of multiple large-scale BFNs derived from rs-fMRI by using a 3-D CNN deep learning architecture for ASD diagnosis. Instead of using 1-D time-series information, we used subject-specific BFN spatial maps to capture the spatial patterns *via* 3-D convolution. Channel attention blocks were integrated into our CNN model to fuse the deep features of multiple BFNs. We evaluated our proposed method on a public dataset and achieved the best performance compared with previous classifications (Heinsfeld et al., 2018; Bengs et al., 2020). Our proposed framework can be easily generalized to the diagnosis of other mental illnesses such as Alzheimer’s, schizophrenia and depression.

Our results showed a mean test accuracy of 77.74% in a 10-fold cross validation experiment using the MCSE-VGG deep learning algorithm. In our proposed algorithm, the input data is multi-channel tensor, and each channel corresponds to one subject-specific BFN. Convolutional layers extract the most important spatial information from these BFNs in a hierarchical way with different layers. The SE module can further integrate and recalibrate these spatial features from different BFNs before they are fed into the next transformation. The introduction of the SE module improves the accuracy of the VGG model by ∼8%. Heinsfeld et al. (2018) proposed to use deep autoencoder network for the classification of ASD. They built a multilayer perceptron (MLP) and applied the encoder weights to the MLP. Only the last layer of MLP was adjusted to output the expected classes. They used functional connectivity features calculated by correlation between each pair of ROIs, which was different from our input features. Some works also used 3-D BFNs as features and developed a 3-D CNN for discrimination of mental illness, e.g., Alzheimer’s dementia (Qureshi et al., 2019b) and schizophrenia (Qureshi et al., 2019a). These studies treated every BFN equally and did not model the contribution of different brain networks to classification results.

The ultimate goal of our proposed framework is to identify a collection of reliable biomarkers for ASD diagnosis. Although deep learning has shown great potential in classification, the lack of interpretability restricts its application in the clinic. To determine the effect of each BFN on the neural network, we used each individual BFN as input features to train a SCSE-VGG model. The results showed that dDMN, PCUN, and SN achieved better accuracies than others. These three brain networks might be the most important features that can boost the performance of MCSE-VGG significantly. Table 6 lists Brodmann areas of these networks.

**TABLE 6:** Anatomical labels of the identified brain functional networks.

BFN	Anatomical location of functional ROIs	Brodmann areas
PCC/MPFC (dDMN)	Medial prefrontal cortex, anterior cingulate cortex, orbitofrontal cortex	9, 10, 24, 32, 11
	Right superior frontal gyrus	9
	Posterior cingulate cortex, precuneus	23, 30
	Midcingulate cortex	23
	Left and right angular gyrus	39
	Left and right thalamus	N/A
	Left and right hippocampus	20, 36, 30
PCUN	Midcingulate cortex, posterior cingulate cortex	23
	Precuneus	7, 19
	Left and right angular gyrus	7, 40
Insula/dACC (SN)	Left middle frontal gyrus	9, 46
	Left and right insula	48, 47
	Anterior cingulate cortex, medial prefrontal cortex, supplementary motor area	23, 32, 8, 6
	Right middle frontal gyrus	46, 9
	Left lobule VI, crus I	N/A
	Right lobule VI, crus I	N/A

Both dDMN and PCUN are subnetworks of the DMN comprising the posterior cingulate cortex (PCC), precuneus, medial prefrontal cortex (MPFC), temporoparietal junction (TPJ), and hippocampus (Raichle et al., 2001; Buckner et al., 2008). The DMN is engaged in a range of social cognitive processes including self-referential and autobiographical processing and mentalizing and theory of mind (Padmanabhan et al., 2017). In particular, the PCC and MPFC, the two most notable nodes of the DMN, are involved with social cognition (Schilbach et al., 2008; Spreng et al., 2009; Mars et al., 2012). The PCC is considered to be the core functional node with a high basal metabolic rate (Raichle et al., 2001). The PCC is mainly engaged in both self-relevant and other-relevant processing and evaluating and processing mental states of others (Gusnard and Raichle, 2001; Schiller et al., 2009; Spreng et al., 2009; Kuzmanovic et al., 2012). The MPFC is linked to monitoring of the mental state of both oneself and others (Gusnard and Raichle, 2001; Schilbach et al., 2008). There is a large amount of literature indicating that abnormal DMN organization is related to social deficits in individuals with ASD (Lynch et al., 2013; Yerys et al., 2015; Ypma et al., 2016). Increased within-network connectivity between the PCC and the MPFC was commonly observed in childhood ASD, while no consistent evidence was found for DMN connectivity across a whole ASD cohort (Doyle-Thomas et al., 2015).

In addition to the DMN and PCUN, the SN has achieved higher classification accuracy at 71.16%. The SN is identified as an intrinsic network that guides behaviors to internal and environmental stimuli (Seeley et al., 2007). The SN exhibited hyper connectivity in children with ASD (Uddin et al., 2013). The anterior insula (AI) and dorsal anterior cingulate cortex (dACC) serve as the two most notable hubs of the SN. The AI is linked to emotion processing and is hypo-activated in ASD cohorts when performing a series of social cognitive tasks (Di Martino et al., 2009). The dACC has showed reduced cognitive control over behavior in ASD (Agam et al., 2010). Restrictive and repetitive behaviors in ASD may be due to the dysfunction of the salience network, which atypically allocates attention to extraneous sensory stimuli rather than relevant social stimuli (Uddin et al., 2013). Our experiments on each individual BFN suggest that the dDMN, PCUN, and SN are highly different between ASD and NC, which is consistent with previous studies. These three most significant brain networks have the potential to be reliable biomarkers for the identification of ASD.

To further validate that our proposed method can capture the interaction between different BFNs, we also considered the relationship between BFNs. A quantitative measure of the connectivity strength value was calculated between each pair of BFNs. The results of statistical analysis suggested that there were significant differences in three pairs of BFNs (at a *p*-value < 0.05). The SN-dDMN and SN-LCEN pairs showed increased connectivity strength in ASD, and hypo-connectivity was observed in the PCUN-dDMN pair of ASD. The SN is thought to play a role in detecting and coordinating a response to salient interoceptive and exteroceptive stimuli, including modulating the DMN and CEN as necessary (Menon, 2011), while aberrant interactions between these networks may lead to various mental illnesses. The enhanced connectivity between the SN and DMN and between the SN and CEN in ASD compared to NC may suggest the aberrant function of the SN, resulting in abnormal saliency mapping of internal mental events and external stimuli. Our work is consistent with the evidence implicating SN dysfunction in psychopathology in ASD (Uddin, 2015; Burrows et al., 2017). Both the PCUN and dDMN include brain regions previously regarded as being part of the DMN (Broyd et al., 2009). The decreased connectivity within the DMN has also been found in previous literature (Assaf et al., 2010). Our findings support the under-connectivity hypnosis in autism proposed by Just et al. (2004). Although no significant differences were found in other pairs of BFNs, the proposed MCSE-VGG is potentially able to capture the interactions among these BFNs, which take both inter- and intra-BFN information into consideration.

Our work still has a few limitations. First, a separate validation dataset is unavailable. The dataset we used is a part of the ABIDE-I dataset, which contained the largest samples with the same scan parameters. However, ABIDE-I is a highly heterogeneous dataset in which the scanning parameters, especially TR and the total scan time of each site, are different from each other. This leads to difficulty in performing group-ICA and dual regression. Yan et al. (2019) developed an RNN framework for discriminating schizophrenia using multi-site fMRI data. However, in their study, the scan protocols were set up by the same experts across all sites, which overcomes the heterogeneity over multi-sites. Therefore, we evaluated our framework only in the NYU dataset and showed ten-fold cross-validation results. Data augmentation has been widely used in many other computer vision tasks, but it is not suitable for neuroimaging data. We do not think synthesized data and real data have the same distribution. Second, motion effects can mimic amplitude effects and may have an impact on the results of dual regression (Nickerson et al., 2017). Motion effects may still remain even though we performed motion correction in the preprocessing procedures. ICA-based denoising technology was proposed to derive reproducible group-level resting state network spatial maps (Pruim et al., 2015), which we will consider using in the preprocessing stage. Another limitation is that deep learning-based classification framework is lacking in interpretation but is important, especially in the medical field. To overcome this issue, we statistically analyzed the differences between each pair of BFNs between ASD and NC. In the future, we will pay more attention to developing a framework integrating both structural and functional MRI data to achieve better classification accuracy, which may provide us with more insights into ASD.

## Conclusion

In conclusion, we proposed a novel framework to model the spatial patterns of BFNs and associations between BFNs, simultaneously. We demonstrated the effectiveness of the introduction of the SE module, which is useful for modeling large-scale brain networks. Aberrant inter-network connectivity was observed in ASD, which may be related to the disorder of brain function. Our work has great application potential in the discrimination of other mental illnesses, not just ASD.

## Data Availability Statement

The original contributions presented in the study are included in the article/supplementary material, further inquiries can be directed to the corresponding author/s.

## Author Contributions

MY and MC had equal contributions in data preparation, data analysis, and preparing the first draft of the manuscript. YuC and YaC interpreted the data and proofread the manuscript. GF and CL put forward some useful suggestions for the project. JW and TL conceptualized, directed the overall project, and prepared the final version. All authors contributed to the article and approved the submitted version.

## Conflict of Interest

The authors declare that the research was conducted in the absence of any commercial or financial relationships that could be construed as a potential conflict of interest.

## Footnotes

1 http://fsl.fmrib.ox.ac.uk/fsl/fslwiki/MELODIC

2 https://fsl.fmrib.ox.ac.uk/fsl/fslwiki/FSLNets

3 http://findlab.stanford.edu/research

4 http://fsl.fmrib.ox.ac.uk/fsl/fslwiki/DualRegression

5 http://fsl.fmrib.ox.ac.uk/fsl/fslwiki/FSLNets
